# Efficient RNA isoform identification and quantification from RNA-Seq data with network flows

**DOI:** 10.1093/bioinformatics/btu317

**Published:** 2014-05-09

**Authors:** Elsa Bernard, Laurent Jacob, Julien Mairal, Jean-Philippe Vert

**Affiliations:** ^1^Mines ParisTech, Centre for Computational Biology, 77300 Fontainebleau, ^2^Institut Curie, 26 rue d'Ulm, 75248 Paris Cedex 05, ^3^INSERM U900, Paris F-75248, France, ^4^Laboratoire Biométrie et Biologie Evolutive, Université de Lyon, Université Lyon 1, CNRS, INRA, UMR5558, Villeurbanne, France and ^5^LEAR Project-Team, INRIA Grenoble Rhône Alpes, 38330 Montbonnot, France

## Abstract

**Motivation:** Several state-of-the-art methods for isoform identification and quantification are based on $\ell_{1}$-regularized regression, such as the Lasso. However, explicitly listing the—possibly exponentially—large set of candidate transcripts is intractable for genes with many exons. For this reason, existing approaches using the $\ell_{1}$-penalty are either restricted to genes with few exons or only run the regression algorithm on a small set of preselected isoforms.

**Results:** We introduce a new technique called FlipFlop, which can efficiently tackle the sparse estimation problem on the full set of candidate isoforms by using network flow optimization. Our technique removes the need of a preselection step, leading to better isoform identification while keeping a low computational cost. Experiments with synthetic and real RNA-Seq data confirm that our approach is more accurate than alternative methods and one of the fastest available.

**Availability and implementation:** Source code is freely available as an R package from the Bioconductor Web site (http://www.bioconductor.org/), and more information is available at http://cbio.ensmp.fr/flipflop.

**Contact:** Jean-Philippe.Vert@mines.org

**Supplementary information:** Supplementary data are available at *Bioinformatics* online.

## 1 INTRODUCTION

Over the past decade, quantitation of mRNA molecules in a cell population has become a popular approach to study the effect of several factors on cellular activity. Typical applications include the detection of genes whose expression varies between two or more populations of samples (differential analysis), classification of samples based on gene expression (van’t Veer *et al.*, 2002) and clustering, which consists of identifying a grouping structure in a sample set (Perou *et al.*, 2000). While probe-based DNA microarray technologies only allow quantitating mRNA molecules whose sequence is known in advance, the recent development of deep sequencing has removed this restriction. More precisely, RNA-Seq technologies (Mortazavi *et al.*, 2008) allow the sequencing of cDNA molecules obtained by reverse transcription of RNA molecules present in the cell. Consequently, any transcript can be sequenced and therefore quantitated, even though its sequence might not be available a priori for designing a specific probe. In addition to facilitating the study of non-coding parts of known genomes and organisms whose genome has not been sequenced (Mortazavi *et al.*, 2010), RNA-Seq technologies facilitate the quantitation of alternatively spliced genes. Genes in eukaryote cells contain a succession of exon and intron sequences. Transcription results in a pre-mRNA molecule from which most introns are removed, and some exons are retained during a processing step called RNA splicing. It is estimated that >95% of multiexonic genes are subject to alternative splicing (Pan *et al.*, 2008): the set of exons retained during splicing can vary, resulting for the same gene in different versions of the mRNA, referred to as transcripts or isoforms. Identification and quantification of isoforms present in a sample is of outmost interest because different isoforms can later be translated as different proteins. Detection of isoforms whose presence or quantity varies between samples may lead to new biomarkers and highlight novel biological processes invisible at the gene level.

Sequencing technologies are well suited to transcript quantitation as the read density observed along the different exons of a gene provide information on which alternatively spliced mRNAs were expressed in the sample, and in which proportions. Because the read length is typically smaller than the mRNA molecule of a transcript, identifying and quantifying the transcripts is, however, difficult: an observed read mapping to a particular exon may come from an mRNA molecule of any transcript containing this exon. Some methods consider that the set of expressed isoforms (Jiang and Wong, 2009) or a candidate superset (Huang *et al.*, 2013; Xing *et al.*, 2006) is known in advance, in which case the only problem is to estimate their expression. However, little is known in practice about the possible isoforms of genes, and restricting oneself to isoforms that have been described in the literature may lead to missing new ones.

Two main paradigms have been used so far to estimate expression at the transcript level while allowing *de novo* transcript discovery. On the one hand, the Cufflinks software package (Trapnell *et al.*, 2010) proceeds in two separate steps to identify expressed isoforms and estimate their abundance. It first estimates the list of alternatively spliced transcripts by building a small set of isoforms containing all observed exons and exon junctions. In a second step, the expression of each transcript is quantified by likelihood maximization given the list of transcripts. Identification and quantification are therefore done independently. On the other hand, a second family of methods (Behr *et al.*, 2013; Bohnert and Rätsch, 2010; Li *et al.*, 2011a, b; Mezlini *et al.*, 2013; Xia *et al.*, 2011) jointly estimates the set of transcripts and their expression using a penalized likelihood approach. These methods model the likelihood of the expression of all possible transcripts, possibly after some preselection, and the penalty encourages sparse solutions that have a few expressed transcripts.

The two-step approach of Cufflinks (Trapnell *et al.*, 2010) is reasonably fast, but does not exploit the observed read density along the gene, which can be a valuable information to identify the set of transcripts. This is a conclusion drawn experimentally using methods from the second paradigm (see Bohnert and Rätsch, 2010; Li *et al.*, 2011a, b; Mezlini *et al.*, 2013; Xia *et al.*, 2011). To summarize, the first paradigm is fast but can be statistically less powerful than the second one in some cases, whereas the second paradigm suffers from the exponential number of candidate isoforms and becomes intractable for genes with many exons. The contribution of this article is to allow $\ell_{1}$-penalized regression methods from the second family to run efficiently without prefiltering the set of isoform candidates, although they solve a non-smooth optimization problem over an exponential number of variables. To do so, we show that the penalized likelihood maximization can be reformulated as a convex cost network flow problem, which can be solved efficiently (Ahuja *et al.*, 1993; Bertsekas, 1998; Mairal and Yu, 2012). Note that a significantly different approach was adopted by Behr *et al.* (2013) for non-convex $\ell_{0}$-penalty. Even though the problem they address is NP-hard, they perform simultaneous isoform identification and quantification, without explicitly enumerating all possible transcripts, by using mixed integer programming techniques.

The article is organized as follows: Section 2 introduces the statistical model (Section 2.1) and the penalized likelihood approach (Section 2.2) we follow. Our model is similar to the one used by Xia *et al.* (2011), but properly models reads that cover more than two exons, effectively taking advantage of longer reads. We then reformulate the model as a path selection problem over a particular graph (Section 2.3), and present our method in Sections 2.4–2.6 called FlipFlop (Fast Lasso-based Isoform Prediction as a FLOw Problem) for solving it efficiently. Section 3 empirically compares our approach with the state of the art on simulated and real sequencing data. Our experiments show that our approach has higher accuracy in isoform discovery than methods that treat discovery and abundance estimation as two separate steps and that it runs significantly faster than methods explicitly listing the candidate isoforms. We discuss the implications of our results in Section 4.

## 2 METHOD

Our approach to isoform deconvolution from RNA-Seq data consists of fitting a sparse probabilistic model, like several existing methods including rQuant (Bohnert and Rätsch, 2010), NSMAP (Xia *et al.*, 2011), IsoLasso (Li *et al.*, 2011b), SLIDE (Li *et al.*, 2011a) or iReckon (Mezlini *et al.*, 2013). The reads from RNA-Seq data are modeled as a linear combination of isoforms expressions that are estimated by using the maximum likelihood principle. Because the number of candidate isoforms grows exponentially with the number of exons, the above methods are either computationally expensive for genes with many exons (such as NSMAP or SLIDE) or include a preselection step to reduce the number of candidates, which may alter the method accuracy.

The main novelty of our article is to tackle the sparse estimation problem efficiently *without prefiltering*. In the methodological section, we show that the corresponding penalized maximum likelihood estimator can be computed in polynomial time with the number of exons, despite the exponential number of candidate transcripts. The key is the use of a non-trivial optimization technique based on the concept of flow in a graph (Ahuja *et al.*, 1993; Mairal and Yu, 2012).

### 2.1 Statistical model

We consider an extension of the model originally introduced by Jiang and Wong (2009) and used in NSMAP for estimating isoform expression for a known set of expressed transcripts. Given a gene of interest, we assume that the list of its *n* exons is known, and that the reads of the RNA-Seq experiments have been mapped to a reference genome. For the purpose of our work, an exon can either be defined by read alignment software as a cluster of reads or from a predefined annotation such as the one provided by the UCSC genome browser (http://genome.ucsc.edu/). In the latter case, exons with alternative 5′-donor and 3′-acceptor sites are considered as two separate exons. For alternative 5′-donor sites, the exon is broken down as one exon ending at the first 5′-donor site, and another one starting at this same point and ending at the second 5′-donor site (similarly for exons with 3′-acceptor sites).

We define a *bin* to be an ordered set of exons. Each read is assigned to a unique bin, corresponding to the exact set of exons that it overlaps. Our model can involve bins with more than two exons. It is thus more general than the one of NSMAP, where bins are simply exons and exon–exon junctions. This extension of NSMAP is particularly useful for long reads, which often cover more than two exons. We summarize the read information by the counts $y_{1},\ldots ,y_{q}$ of reads falling in *q* different bins.

We consider in our model all *m* possible candidate isoforms consisting of an ordered sequence of exons. Each candidate isoform corresponds to a unique sequence of bins. This sequence can be generated by virtually moving a read along the candidate isoform, and recording the sets of exons that it successively overlaps.

The *effective length l_i_* of a bin *i* is defined as the number of positions in the candidate isoform where reads can start and be assigned to the bin. A simple computation shows that for a bin involving a single exon of length *l_e_*, we have $l_{i}=l_{e}-L+1$, where *L* is the read length, whereas for bins involving several exons, $l_{i}=\min(l_{\text{left}},L-l_{\text{int}}-1)+\min(l_{\text{right}},L-l_{\text{int}}-1)-L+l_{\text{int}}+1$, where $l_{\text{left}}$ and $l_{\text{right}}$ are the lengths of the leftmost and rightmost exons of the bin, and $l_{\text{int}}$ is the total length of the internal exons of the bin. Interestingly, we note that the effective length of a bin does not depend on the candidate isoform it is associated with. A figure illustrating the computation of the effective length is given in Section 1 of the Supplementary Material.

We model read counts as independent Poisson random variables whose means are proportional to the bins’ effective lengths and to the total abundances of isoforms associated with each bin. More formally, let us denote by *U* the m×q binary matrix defined as $U_{ji}=1$ if bin *i* is associated to isoform *j* and 0 otherwise, and by $\theta_{j}\in {\mathbb{R}}_{+}$ the expression of isoform *j* (the expected number of reads per base in isoform *j*). Thus, $\sum_{j=1}^{m}U_{ji}\theta_{j}$ represents the sum of expressions of all isoforms involving bin *i*. We expect the observed count for bin *i* to be distributed around this value times the effective length of the bin *l_i_*, and therefore model the read count $y_{i}$ as a Poisson random variable with parameter $\delta_{i}=l_{i}\sum_{j=1}^{m}U_{ji}\theta_{j}$. For a vector $\theta ={\left[\theta_{j}\right]}_{j=1}^{m}$ in ${\mathbb{R}}_{+}^{m}$, this yields the log-likelihood

(1) $$L(\theta )=\sum_{i=1}^{q}\left[-\delta_{i}+y_{i}\log\delta_{i}-\log(y_{i}!)\right]$$

where the scalars $\delta_{i}$ depend linearly on θ.

Maximizing the likelihood (1) allows quantifying the relative abundance of each transcript when the model only includes the list of ‘true’ isoforms present in the sample (Jiang and Wong, 2009). Because this list is unknown a priori, we present in the next section the sparse estimation approach that can jointly quantify and identify the transcripts using all candidate isoforms, following Xia *et al.* (2011).

### 2.2 Isoform detection by sparse estimation

Because we do not assume that the list of expressed isoforms—i.e. such that $\theta_{j}\neq 0$—is known in advance, we endow θ with an exponential prior $\theta_{j}\overset{\text{iid}}{∼}E(\lambda )$ and maximize over all candidate isoforms the resulting posterior likelihood, leading to the estimator
(2)
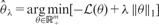

where λ is a regularization parameter, and the $\ell_{1}$-norm is defined as ${\left|\theta \right|}_{1}=\sum_{j=1}^{m}|\theta_{j}|$. It is well-known that the $\ell_{1}$-norm penalty and the non-negativity constraint have a sparsity-inducing effect—that is, lead to estimators ${\hat{\theta }}_{\lambda }$ that contain many zeroes (Tibshirani, 1996). The parameter λ controls the number of non-zero elements in the solution ${\hat{\theta }}_{\lambda }$, i.e. of selected isoforms, with larger λ corresponding to fewer isoforms.

Mezlini *et al.* (2013) claim that the $\ell_{1}$-penalty is inappropriate for isoform selection, a claim we disagree with. As they note, the sum of true abundances in RPKM weighted by isoform lengths is by definition the true proportion of reads coming from the gene times 10^9^. They conclude that penalizing by $\sum_{j}\theta_{j}$ has little effect on the estimate. However, the sum of the *estimator* ${\hat{\theta }}_{\lambda }$ weighted by isoform lengths has no reason to be equal to the *observed* number of reads mapping to the gene. There are several causes for that: model inadequacy, various noise sources, finite sample size and bias of the estimator. Penalizing this sum therefore modifies the sparsity level of ${\hat{\theta }}_{\lambda }$ as observed in our and other’s experiments (Bohnert and Rätsch, 2010; Li *et al.*, 2011a, b; Xia *et al.*, 2011).

Note also that (2) is better adapted to long reads than the original formulation of NSMAP (Xia *et al.*, 2011), thanks to the use of general bins. rQuant (Bohnert and Rätsch, 2010), IsoLasso (Li *et al.*, 2011b) and SLIDE (Li *et al.*, 2011a) solve a similar problem where the likelihood is a simpler quadratic function, corresponding to a Gaussian model for the read counts. A difficulty with these approaches is that the dimension *m* grows exponentially in *n*, making (2) intractable when *n* is large. For example, Li *et al.* (2011a) restrict themselves to experiments involving genes with <10 exons, because of the high computational cost for larger genes. Xia *et al.* (2011) restrict themselves to genes with <80 exons, but only consider candidates with transcription start/polyadenylation sites (TSS/PAS) pairs already observed in annotations, and which involve more than half of the exons of the gene. Other approaches such as IsoLasso include a filtering step to reduce the number of isoforms, similarly as Cufflinks does—in the case of single-end reads, their set of candidates is the set of isoforms returned by Cufflinks. As pointed out in Section 1, this filtering may lead to a loss of power in isoform detection because it disregards the read density information when constructing the set of candidates. In the next section, we show that, surprisingly, problem (2) can be solved efficiently without prefiltering the isoforms by using network flow algorithms.

### 2.3 Isoform detection as a path selection problem

In this section, we reformulate the isoform detection problem as a path selection problem over a particular graph. Remember that a graph G=(V,E) is composed of a finite set of vertices *V* and edges E⊆V×V. A path is a sequence of vertices $v_{1},\ldots ,v_{k}\in V$ such that $\left(v_{i},v_{i+1}\right)$ is an arc in *E* for all indices 1≤i<k. A graph is a directed acyclic graph (DAG) if it contains no path $\left(v_{1},\ldots ,v_{k}\right)$ with $v_{1}=v_{k}$. In other words, the graph does not contain any cycle.

**Fig. 1.**
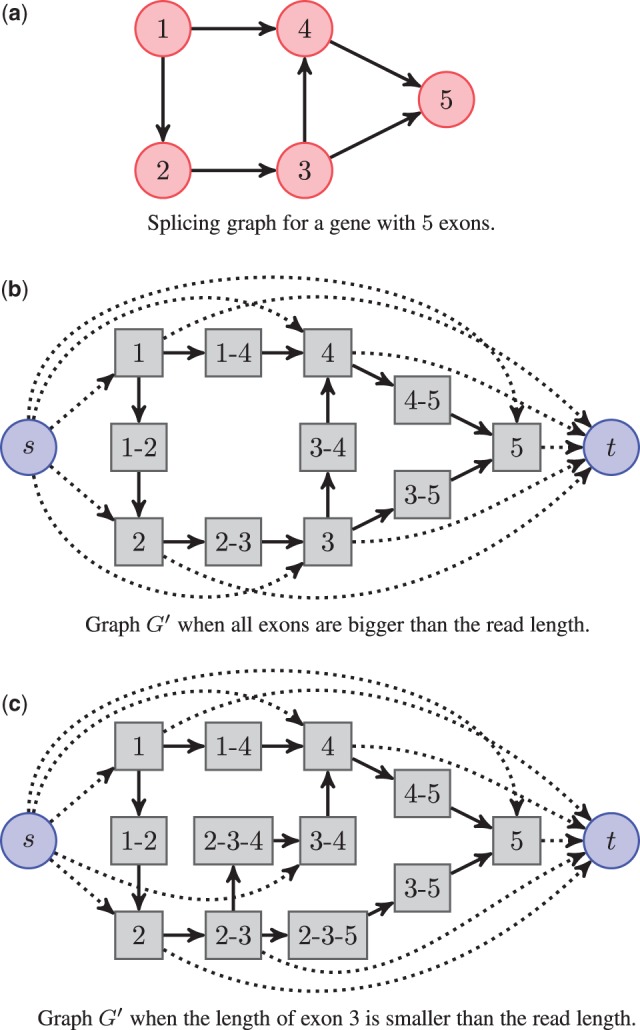
Illustration of the graph construction for a gene with 5 exons. The original splicing graph is represented in (**a**). The 5 exons are represented as vertices and an arrow between two vertices indicates a junction. The nodes of graph G′ in (**b**) and (**c**) are bins with positive effective length denoted by gray square, as well as source *s* and sink *t* represented as circles. G′ in (b) is the resulting graph when all exons are bigger than the read length. In that case, each bin either corresponds to a unique exon, or to a junction between two exons. G′ in (c) is the resulting graph when the length of exon 3 is smaller than the read length. Some bins involve then more than two exons, here bins (2-3-4) and (2-3-5). The source links all possible starting bins, and conversely all possible stopping bins are linked to the sink. There is a one-to-one correspondence between (*s*, *t*)-paths in G′ (paths starting at *s* and ending at *t*) and isoform candidates. For example, the path (s,1,1-4,4,4-5,5,t) corresponds to isoform 1-4-5

We construct an oriented graph G=(V,E) whose vertices are the bins with positive effective length defined in Section 2.1—each corresponding to an ordered set of exons. An edge connects bin *v_i_* to *v_j_* if they can be associated to two reads starting at successive positions in a candidate isoform. In general, *v_j_* is obtained from *v_i_* by removing the first exon of its ordered set or by adding one extra exon at the end of the ordered set, depending on the lengths of the exons composing the bin (Fig. 1). We call *starting bins* (respectively *stopping bins*) the bins that can contain a read at the left-most (respectively right-most) position of an isoform. The resulting graph is a DAG generalizing the splicing graph (Heber *et al.*, 2002), whose vertices are single exons and edges are exon–exon junctions.

We also consider two new vertices *s* and *t*, respectively, dubbed *source* and *sink*, and connected to all starting and stopping bins, respectively. We do not impose any restriction on the set of TSS/PAS sites and each exon can potentially start or end an isoform. Consequently, the source is connected to all bins modeling an exon start, and the sink to all bins modeling an exon end. This leads to the definition of an extended graph G′=(V′,E′) with V′=V∪{s,t} and E′ is obtained by adding to *E* all edges of the form (*s*, *v*) where v∈V is a starting bin, and (*v*, *t*) where v∈V is a stopping bin. This graph construction is illustrated in Figure 1. Montgomery *et al.* (2010) use a similar graph structure in the context of estimating the expression of a set of known annotated transcripts.

Let us denote by P the set of paths in G′ starting from *s* and ending at *t*, which are called (*s*, *t*)-paths. By construction, one easily checks that P is in bijection with the set of candidate isoforms, the path in G′ corresponding to a candidate isoform being the set of bins that reads generated from the isoform can produce. Based on this one-to-one mapping, we can reformulate the penalized maximum likelihood problem (1)–(2) as follows: we want to find non-negative weights $\theta_{p}$ for each path p∈P which minimize:
(3)
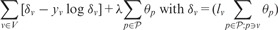

where the sum 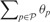 is equal to the $\ell_{1}$-norm $||\theta |{|}_{1}$ because the entries of θ are non-negative. Note that we have removed the constant term $\log(y_{v}!)$ from the log likelihood because it does not play a role in the optimization. This reformulation is therefore a path selection (finding which $\theta_{p}$ are non-zero) and quantification problem over G′. The next section shows how (3) can further be written as a flow problem, i.e. technically a constrained optimization problem over the edges of the graph rather than the set of paths in P. A computationally feasible approach can then be devised to solve (3) efficiently, following Mairal and Yu (2012).

### 2.4 Optimization with network flows

A *flow f* on G′ is defined as a non-negative function on arcs ${\left[f_{uv}\right]}_{(u,v)\in E\prime }$ that satisfies conservation constraints: the sum of incoming flow at a vertex is equal to the sum of outgoing flow except for the source *s* and the sink *t*. Such conservation property leads to a physical interpretation about flows as quantities circulating in the network, for instance, water in a pipe network or electrons in a circuit board. The source node *s* injects into the network some units of flow, which move along the arcs before reaching the sink *t*.

For example, given a path p∈P and a non-negative number $\theta_{p}$, we can make a flow by setting $f_{uv}=\theta_{p}$ when *u* and *v* are two consecutive vertices along the path *p*, and *f_uv_* = 0 otherwise. This construction corresponds to sending $\theta_{p}$ units of flows from *s* to *t* along the path *p*. Such simple flows are called (*s*, *t*)*-path flows*. More interestingly, if we have a set of non-negative weights $\theta \in {\mathbb{R}}_{+}^{\left|P\right|}$ associated to all paths in P, then we can form a more complex flow by superimposing all (*s*, *t*)-path flows according to
(4)
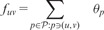

where (u,v)∈p means that *u* and *v* are consecutive nodes on *p*.

**Fig. 2.**
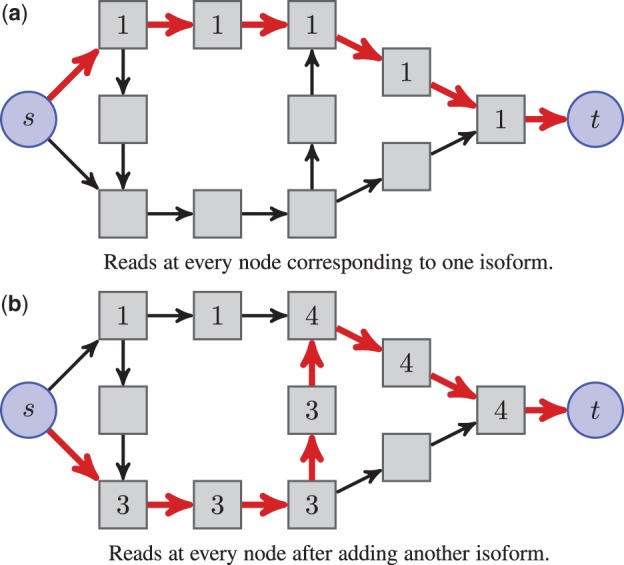
Flow interpretation of isoforms using the same graph as in Figure 1b. For the sake of clarity, some edges connecting *s* and *t* to internal nodes are not represented, and the length of the different bins is assumed to be equal. In (**a**), one unit of flow is carried along the path in red, corresponding to an isoform with abundance 1. In (**b**), another isoform with abundance 3 is added, yielding additional read counts at every node

While (4) shows how to make a complex flow from simple ones, a converse exists, known as the *flow decomposition theorem* (see, e.g. Ahuja *et al.*, 1993). It says that for any DAG, every flow vector can always be decomposed into a sum of (*s*, *t*)-path flows. In other words, given a flow ${\left[f_{uv}\right]}_{(u,v)\in E\prime }$, there exists a vector θ in ${\mathbb{R}}_{+}^{\left|P\right|}$ such that (4) holds. Moreover, there exist linear-time algorithms to perform this decomposition (Ahuja *et al.*, 1993). As illustrated in Figure 2, this leads to a flow interpretation for isoforms.

We now have all the tools in hand to turn (3) into a flow problem by following Mairal and Yu (2012). Given a flow 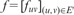, let us define the amount of flow incoming to a node *v* in *V*′ as 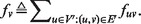 Given a vector 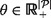 associated to *f* by the flow decomposition theorem, i.e*.* such that (4) holds, we remark that 
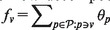
 and that 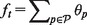. Therefore, problem (3) can be equivalently rewritten as
(5)
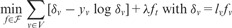

where F denotes the set of possible flows. Once a solution f* of (5) is found, a solution θ* of (3) can be recovered by decomposing f* into (*s*, *t*)-path flows, as discussed in the next section.

The use of network flows has two consequences. First, (5) involves a polynomial number of variables, as many as arcs in the graph, whereas this number was exponential in (3). Second, problem (5) falls into the class of *convex cost flow* problems (Ahuja *et al.*, 1993), for which efficient algorithms exist [The function (5) can be decomposed into costs $C_{v}(f_{v})$ over vertices *v*. The general convex cost flow objective function is usually presented as a sum of costs $C_{uv}(f_{uv})$ over arcs (*u*, *v*). It is however easy to show that costs over vertices can be reduced to costs over arcs by a simple network transformation (see Ahuja *et al.*, 1993, Section 2.4). Note that all arcs have zero lower capacities and infinite upper capacities.]. In our experiments, we implemented a variant of the scaling push-relabel algorithm (Goldberg, 1997), which also appears under the name of ε-relaxation method (Bertsekas, 1998). Note that the approach can be generalized to any concave likelihood function, including the Gaussian model used by IsoLasso and SLIDE.

Network flows have been used in several occasions in bioinformatics. Medvedev and Brudno (2009) solve a convex cost flow problem on a bidirected de Bruijn graph for maximum likelihood whole genome shotgun assembly. Montgomery *et al.* (2010) introduced the formalism of flows for RNA-Seq data; however, they did not perform isoform discovery but quantification from a set of known transcripts. Their formulation is a linear program, the dimension of which is the number of candidate transcripts considered, which is not a network flow problem. Singh *et al.* (2011) uses the terminology of flows for RNA-Seq data in the context of testing differential transcription without reconstructing transcripts. Finally, while this article was under revision, Tomescu *et al.* (2013) published a similar method, which also uses minimum cost flow techniques for isoform recovery. However, their method only involves bins corresponding to exons and exon–exon junction and, more importantly, does not solve the *penalized* likelihood approach. They have therefore no principle way to balance the sparsity of the solution with its likelihood, and even mention that this leads to a NP-hard problem. To our knowledge, our work is the first to show that the sparsity-inducing $\ell_{1}$ penalty can be integrated with the likelihood term in the language of network flow, to estimate a flow with large likelihood that can be easily decomposed in a number of paths as small as we wish.

### 2.5 Flow decomposition

We have seen that after solving (5) we need to decompose f* into (*s*, *t*)-path flows to obtain a solution θ* of (2). As illustrated in Figure 2, this corresponds to finding the two isoforms from 2(b). Although the decomposition might not be ambiguous when f* is a sum of few (*s*, *t*)-path flows, it is not unique in general. Our approach to flow decomposition consists of finding an (*s*, *t*)-path carrying the maximum amount of flow (equivalently finding an isoform with maximum expression), removing its contribution from the flow and repeating until convergence. We remark that finding (*s*, *t*)-path flows according to this criterion can be done efficiently using dynamic programming, similarly as for finding a shortest path in a directed acyclic graph (Ahuja *et al.*, 1993). We insist on the fact that the flow decomposition returns one solution of the ℓ_1_-penalized estimator given by problem 3. This problem can have several solutions yielding the same objective value, and which are typically sparse in the number of transcripts (see Supplementary Fig. S2). The non-uniqueness of the solution is not an artifact of our network flow approach, but a property of the ℓ_1_-penalized estimator. Algorithms such as SLIDE, NSMAP or others that explicitly enumerate the candidates and minimize the parameter in the candidate space also return one of several solutions. In parallel, the stability of the estimator is investigated in Section 4 of the Supplementary Material.

### 2.6 Model selection

The last problem we need to solve is model selection: even if we know how to solve (2) efficiently, we need to choose a regularization parameter λ. For large values of λ, (2) yields solutions involving few expressed isoforms. As we decrease λ, more isoforms have a non-zero estimated expression $\theta_{j}$, leading to a better data fit but also leading to a more complex model. A classical way of balancing fit and model complexity is to use likelihood ratio tests. Xia *et al.* (2011) chose this approach, but we found the log likelihood ratio statistics to be empirically poorly calibrated because of the typically small number of samples units—exons—and the non-independence of the observed read counts. We choose a related approach, which we found better behaved, and select the model having the largest bayesian information criterion (BIC) (Schwarz, 1978). An alternative approach taken by Li *et al.* (2011a) would be to use stability selection (Meinshausen and Bühlmann, 2010).

## 3 RESULTS

We now compare our proposed method FlipFlop to Cufflinks (Trapnell *et al.*, 2010) version 2.0.0, IsoLasso (Li *et al.*, 2011b) version 2.6.1 and NSMAP (Xia *et al.*, 2011) on both simulated and real data. All experiments were run on a desktop computer on a single core of an Intel Xeon CPU X5460 3.16 Ghz with 16 GB of RAM. Reads are aligned to a reference genome by using TopHat (Trapnell *et al.*, 2009) version 2.0.6, and the constructed alignment files are used as input to the methods we compare. IsoLasso, Cufflinks and FlipFlop only use these aligned reads as input, and estimate their exon boundaries and TSS/PAS from read density. NSMAP additionally requires exon boundaries and known TSS/PAS as input. For paired-end experiments, we extended our initial model designed for single-end: a pair of reads is considered as a long single-end read. When the two reads of a pair span bins potentially separated by some exons, we use heuristics based on genomic distances to decide whether these exons are spliced. All software programs are used with default parameters, except that for paired-end experiments, we provide fragment length mean and standard deviation to IsoLasso, Cufflinks and FlipFlop. Note that all results can be easily reproduced by following the tutorials available at http://cbio.ensmp.fr/flipflop/experiments.html.

### 3.1 Simulated human RNA-Seq data

Because little is known about the true set of isoforms expressed in real data, we start our experimental validation with a set of simulations. We use the RNASeqReadSimulator software (http://alumni.cs.ucr.edu/∼liw/rnaseqreadsimulator.html) to generate single-end and paired-end reads from the annotated human transcripts available in the UCSC genome browser (hg19). We restrict ourselves to the 1137 multi-exons genes on the positive strand for chromosome 1, corresponding to 3709 expressed transcripts.

We follow the protocol of Isolasso (Li *et al.*, 2011b) and consider that a transcript from the annotation has been detected by a method if it predicts a transcript that (i) includes the same set of exons, and such that (ii) all internal boundary coordinates (i.e. all the exon coordinates except the beginning of the first exon and the end of the last exon) are identical. The objective for each method is to recover a large proportion of transcripts that were used for read generation—high recall—without detecting too many transcripts that were not used to generate the reads—high precision.

**Fig. 3.**
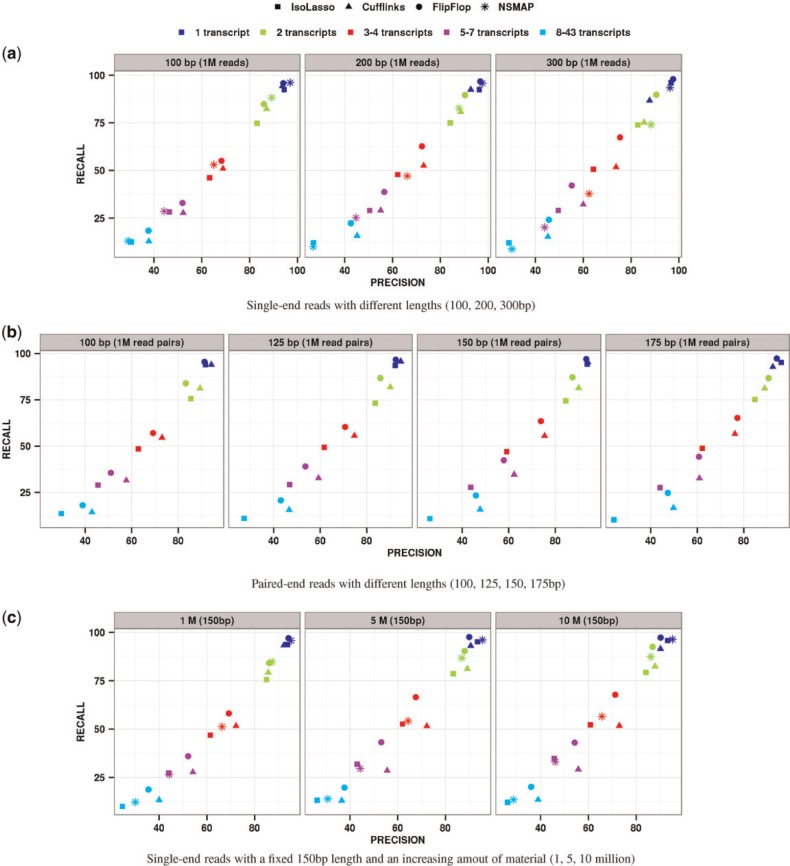
Precision and recall of compared methods on simulated reads from the UCSC annotated human transcripts. (a) Single-end reads with different lengths (100, 200, 300 bp). (b) Paired-end reads with different lengths (100, 125, 150, 175 bp). (c) Single-end reads with a fixed 150 bp length and an increasing amout of material (1, 5, 10 million)

Figure 3 shows the precision and recall of the compared methods on single-end and paired-end simulations. Because we expect the difficulty of the deconvolution problem to increase with the number of transcripts of the gene, we stratify the result by this number: each dot represents the precision and recall of one method for genes with a particular number of transcripts in the UCSC annotation. As expected, genes with more transcripts lead to more difficult estimation problems and decreased performances for all methods. Figure 3a shows single-end results for different read lengths from 100 to 300 bp and a fixed number of 1 million reads per experiment. FlipFlop clearly takes advantage of longer reads: the longer the read the better the accuracy for all transcript levels. For 100 bp long reads, FlipFlop and Cufflinks show similar results, while NSMAP gives slightly better precision and recall for two transcript level and degraded results compared with FlipFlop for more than four expressed transcripts. These differences might arise from the fact that NSMAP restricts its search to the TSS and PAS observed in the annotation, whereas FlipFlop estimates them from reads, and the fact that the two methods use different graphs and model selection techniques. For 300 bp long reads, FlipFlop outperforms all other methods as soon as there is more than one expressed transcript. For instance, for the three to four transcripts levels, FlipFlop achieves 75% of precision and 67% of recall, while Cufflinks reaches 74 and 52% and IsoLasso reaches 64 and 51%. This demonstrates that an adapted model for long reads is critical for isoform recovery. NSMAP optimizes a similar Poisson objective function as FlipFlop but only models reads at the exon or exon–exon junction levels; it looses statistical power when the read length increases. Figure 3b shows paired-end results for 400 bp fragment length, 20 bp SD, 1 million read pairs and read lengths from 100 to 175 bp. Although our model is designed for single-end reads and is particularly adapted to long reads, it shows competitive or better accuracy for paired-end reads. Once again, when the read length increases, FlipFlop performance improves proportionally more than other methods. In Figure 3c, the read length is set to 150 bp and the number of simulated reads varies from 1 million to 10 million. Increasing the coverage clearly helps FlipFlop, whereas it does not change much for Cufflinks and IsoLasso. Cufflinks constructs its set of transcripts and estimates their abundances in two separate steps, and the construction of the set of returned transcripts does not take read density into account: it intends to find the smallest set of isoforms covering all the observed reads. IsoLasso is based on penalized likelihood maximization like FlipFlop and NSMAP, but starts from a restricted set of isoforms—the same set returned by Cufflinks for single-end data. Consequently, this family of methods discards some information that can help identifying the set of expressed isoforms.

While results in Figure 3 are obtained with default parameters for all methods, Supplementary Figure S6 of the supplementary shows performances when parameters are first tuned on an independent training set; in that case, results are not significantly different. Note that the number of exons of a gene significantly affects the difficulty of isoform reconstruction. We show in the Supplementary Material (Supplementary Fig. S3) similar results to the ones presented in Figure 3 with stratification by number of exons instead of number of transcripts. We also detail in Section 6 of the Supplementary Material more realistic simulations that include typical library preparation and sequencing biases using another simulator—the Flux-Simulator (Griebel *et al.*, 2012), which aims at modeling RNA-Seq experiments *in silico*—and we show results that are consistent with the ones of Figure 3.

**Fig. 4.**
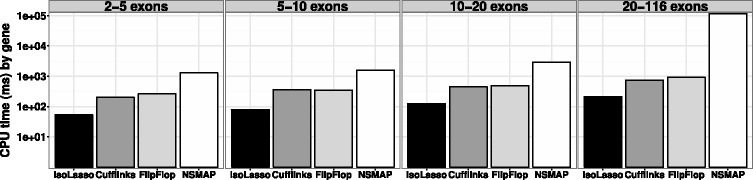
Average CPU times in milliseconds (logarithmic scale) for the compared methods with process a gene from human simulated 100 bp single-end reads

Figure 4 shows the mean CPU time taken by each method to perform the deconvolution of genes with different sizes. Genes with more exons tend to have more candidate isoforms and experiments involving such genes are expected to take more time. Therefore, we stratify the observed times by exon number of the genes: each barplot represents the mean time taken by each method for finding the transcripts of genes having a particular number of exons. As expected, FlipFlop is always faster than NSMAP, more than a hundred times faster for genes with >20 exons. FlipFlop speed is comparable with Cufflinks, and about four times slower than IsoLasso. This is because IsoLasso maximizes its objective over a very restricted set of candidates—in these simulations never more than nine and around two to three on an average. Overall, FlipFlop estimates the set of expressed isoforms for 1137 genes in <9 min, i.e. about two genes per second. Note also that the time for data pre-preprocessing (finding exon boundaries and read counts for exons and junctions) is taken into account for all methods except NSMAP.

These simulations confirm several facts. First, methods that identify and quantify transcripts as a single penalized maximum likelihood problem show good performances and take clear advantage of an increase in coverage. Second, correctly modeling long reads allows to greatly improve the accuracy of isoform reconstruction. Third, the proposed network flow strategy allows to solve the penalized likelihood approach quickly even when the set of candidate isoforms is extremely large.

### 3.2 Real RNA-Seq data

Our second round of experiments involves two independent real human embryonic stem cell datasets. They both contain about 50 million 75 bp reads, either paired-end or single-end, with respectively NCBI SRA accession number SRR065504 and ERR361241.

In the experiments of Section 3.1, we generated the reads based on a known set of transcripts. In the present case, the reads come from actual human tissues, and we do not have access to the true set of expressed transcripts. Following Xia *et al.* (2011) and Li *et al.* (2011a), we choose to use the UCSC annotation as ground truth in the evaluation. Admittedly, this is not perfect as some expressed transcripts may be missing from the annotation, and some annotated transcripts may not be expressed in this particular experiment. However, agreement of the prediction with the set of known transcripts could be a good sign.

**Fig. 5.**
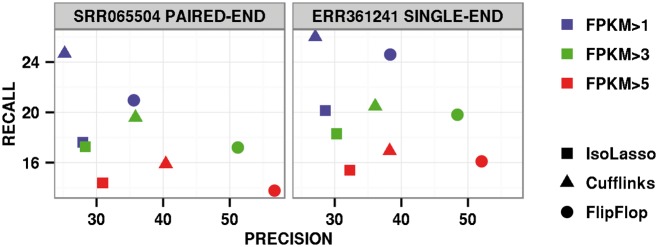
Precision and recall of compared methods on human embryonic stem cells data

Figure 5 shows precision and recall of each method for different FPKM (Fragments Per Kilobase of exon per Million fragments mapped) levels. When considering all transcripts with predicted abundances higher than 1 FPKM, FlipFlop has a higher precision for both the paired-end and single-end datasets, while Cufflinks has a better recall. For transcripts with more than 5 FPKM abundance, all methods have a similar recall, with a slight advantage to Cufflinks, while FlipFlop shows a much better precision. Section 7 of the supplementary gives additional details on the real RNA-Seq data experiments: Supplementary Figure S8 shows the running time of the compared methods and Supplementary Figure S9 corresponds to the precision-recall curves obtained for FlipFop when varying the model selection rule.

## 4 DISCUSSION

Simultaneously tackling identification and quantitation using penalized likelihood maximization is known to be a powerful approach to estimate the set of expressed transcripts. However, existing $\ell_{1}$-regularized regression techniques cannot deal with genes that contain too many exons, as the set of candidate isoforms grows exponentially with the number *n* of exons. By leveraging network flow optimization algorithms, we discover a few expressed transcripts among the exponential number of candidates by solving a problem with a number of variables polynomial in *n*.

We compared our approach to existing $\ell_{1}$-penalized likelihood maximization methods as well as methods that define expressed isoforms as the smallest set of transcripts covering all observed reads; the latter methods perform abundance estimation in a separate step. We observed on simulation data—where the true set of expressed transcripts is known—that, unlike the second set of methods, penalized likelihood maximization methods take advantage of an increase in read coverage. Moreover, we show that correctly modeling long reads is of primary importance for isoform recovery. Our approach, which models reads covering any number of exons, outperforms other methods for 300 bp long reads. We believe this is an important improvement as RNA-Seq technologies are moving forward longer reads. Our FlipFlop method has also shown to be competitive with state-of-the-art methods on real single-end and paired-end human stem cells data, especially for transcripts whose abundance was significant. In addition, the runtime of our method was comparable with the runtime of the second set of methods, and orders of magnitude faster than existing software for penalized likelihood maximization.

We believe these results have important practical implications. In addition to the obvious gain in time when estimating the expression of transcripts for a single gene and a single sample, our approach makes the task amenable in a reasonable amount of time for all genes in a large number of samples. This is a necessary step for high-throughput differential expression studies at the transcript level, a direction we are planning to explore in future work. Differential expression studies were until now restricted to gene level studies, i.e. ignoring the transcript level information, to cases where the set of expressed transcripts was known in advance or to methods which were not using the read density to estimate the set of expressed transcripts—a less efficient approach as illustrated in our experiments. Furthermore, accurately estimating the transcript-level expression for all genes of all samples in a study may lead to improvements in molecular based diagnosis or prognosis tools, as well as in clustering of samples, e.g for cancer subtype discovery. The ability of our approach to deal with splicing graphs with potentially hundreds of nodes also paves the way to efficient *de novo* transcript identification, where we do not restrict ourselves to annotated exons within a single gene.

## Supplementary Material

Supplementary Data
