# Supplementary material for: Efficient RNA isoform identification and quantification from RNA-Seq data with network flows
Source: Bioinformatics. 2014 May 9;30(17):2447–55. doi: 10.1093/bioinformatics/btu317 (PMC4147886; doi:10.1093/bioinformatics/btu317)
Supplement: Supplementary Data [file supp_30_17_2447__index.html]

Efficient RNA isoform identification and quantification from RNA-Seq data with network flows — Efficient RNA isoform identification and quantification from RNA-Seq data with network flows — Supplementary Data 

# Efficient RNA isoform identification and quantification from RNA-Seq data with network flows

## Supplementary Data

files

**Files in this Data Supplement:**

- Supplementary Data - pdf file
