# Supplementary material for: Efficient RNA isoform identification and quantification from RNA-Seq data with network flows
Source: Bioinformatics. 2014 May 9;30(17):2447–55. doi: 10.1093/bioinformatics/btu317 (PMC4147886; doi:10.1093/bioinformatics/btu317)
Supplement: Supplementary Data [file supp_btu317_Bernard_Supp_IsoformFlows.pdf]

BIOINFORMATICS

Vol. 00 no. 00 2013
Pages 1–5

Supplementary Material for the *Bioinformatics* submission "Efficient RNA Isoform Identification and Quantification from RNA-Seq Data with Network Flows"

Elsa Bernard,[1,2,3], Laurent Jacob,[4], Julien Mairal,[5], Jean-Philippe Vert,[1,2,3]

[1]Mines ParisTech, Fontainebleau, France, [2]Institut Curie, Paris, France, [3]INSERM U900, Paris, France, [4]LBBE, Lyon, France, [5]LEAR Project-Team, INRIA Grenoble - Rhône Alpes, France

Received on XXXXX; revised on XXXXX; accepted on XXXXX

Associate Editor: XXXXXXX

1 BINS AND EFFECTIVE LENGTHS

We defined in section 2.1 of the main paper a *bin* as an ordered set of exons that can contain a read with a given length L. We also defined the *effective length* l_i of a bin i as the number of available positions in the bin where a read can start and be assigned to the bin (a read is assigned to a bin if it overlaps all the exons of the bin and is contained by it).

Figure S1 helps to understand this definition. We schematize a bin with two exons of lengths l_{left} and l_{right}, and such that $l_{\text{left}}+l_{\text{right}} \geq L$ (hence the two exons can indeed contain a read and the bin is valid). The red marks correspond to the positions where a read can start and be assigned to the bin. There are four possible cases depending of the relative order of the lengths l_{left}, l_{right} and L: when both l_{left} and l_{right} are bigger than L, the effective length only depends of the read length ($l_i = L - 1$), when only one of the exons is strictly smaller than the read length then the effective length equal the length of that exon ($l_i = l_{\text{left}}$ or $l_i = l_{\text{right}}$), and when both exons are strictly smaller than L, the effective length is equal to $l_{\text{left}} + l_{\text{right}} - L + 1$. These four cases for a multi-exons bin can be written in a single formula: $l_i = \min(l_{\text{left}}, L-1) + \min(l_{\text{right}}, L-1) - L + 1$. Note that when a bin is composed of more than two exons, the reasoning is the same by replacing the read length L by $L - l_{\text{int}}$ where l_{int} is the total length of the internal exons of the bin.

2 SPARSITY OF THE ℓ_1-PENALIZED ESTIMATOR

We illustrate here the fact that the flow decomposition returns a solution of the ℓ_1-penalized estimator (problem 3 in the main paper) which is sparse in the number of transcripts. Figure S2 shows the final number of predicted transcripts after flow decomposition and model selection for genes with a particular number of expressed transcripts.

3 GENE SIZE INFLUENCE ON ISOFORM RECOVERY

In the main paper we stratified precision and recall for isoform recovery by the number of expressed transcripts for each gene (Figure 3) . The number of exons of a gene is also a parameter that affects greatly the difficulty of the problem. Indeed, the more exons the bigger the set of candidate transcripts. Figure S3 shows similar experiments as the ones presented in Figure 3 of the main paper with the only difference being the exon stratification instead of the transcript stratification. The number of exons varies from 2 to 116 and we compare FlipFlop, Cufflinks and IsoLasso.

For both single-end and paired-end reads, FlipFlop performance increases greatly compared to Cufflinks and IsoLasso when the read length increases (Figure S3(a) and Figure S3(b)). For 300bp read length FlipFlop outperforms Cufflinks and IsoLasso for all genes with between 2 and 20 exons. Similarly to what we observed on simulations by transcript levels, and because FlipFlop predicts its transcripts by using both read alignment positions and read density without any filtering, an increase in coverage leads to better results for all exon levels (Figure S3(c)).

4 STABILITY STUDY

We study in detail the stability of the proposed approach, *i.e.*, how a solution is affected by some small perturbations of the input data. We first emphasize that, in some cases, several set of isoforms of the same size might explain equally well a set of RNA-Seq reads. Consider for instance a three exons gene A-B-C with twice more reads mapping into exon B that in exons A and C. In that case solutions (A-B, B-C) and (A-B-C, B) explain equally well the mapped reads. This non-unicity problem is important but can not be solved by the ℓ_1-criteria, nor the ℓ_0, which will both return one of the two solutions. We check empirically in what extend the solutions at a given locus might be ambiguous. For a given coverage we simulated independently two sets of reads from UCSC annotated human transcripts. This corresponds to simulate biological replicates. We then performed the isoform deconvolution for the two sets and defined the stability as the percentage of common isoforms between the two solutions. This procedure was repeated ten times for each coverage. Results are shown in Figure S4 for Cufflinks and FlipFlop. For both methods the stability decreases with the number of expressed transcripts and increases with coverage. Overall Cufflinks becomes approximately 10% more stable than FlipFlop for high coverage for genes with multiple transcripts. The stability results should be of course put in perspective with the accuracy of the isoform deconvolution.

© Oxford University Press 2013.

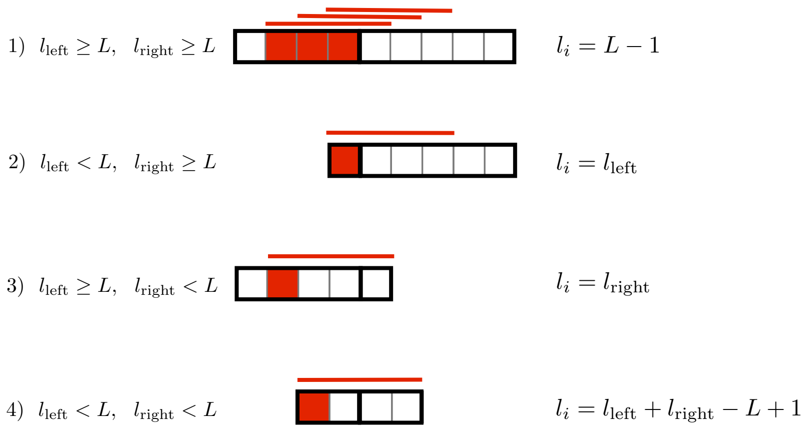

Fig. S1. Computation of the effective length l_i. Here the bin is composed of two exons of lengths l_{left} and l_{right}, drawn in solid black line. Red lines represent the reads of length L. The red squares correspond to the position where a read can start and be assigned to the bin.

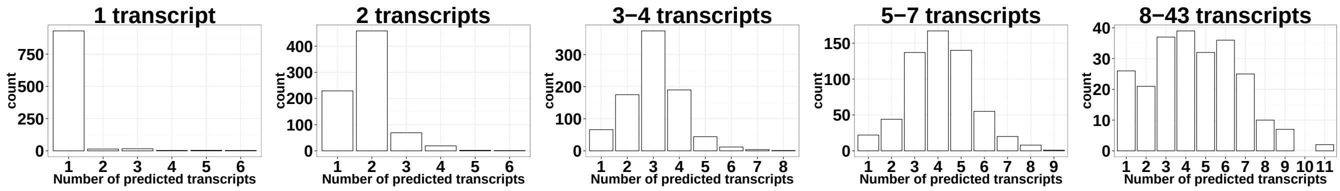

Fig. S2. Number of predicted transcripts for human RNA-Seq simulations with 150bp long single-end reads and 1 million reads by expressed transcript levels.

5 TUNING PARAMETERS

The simulations presented in section 3.1 of the main paper correspond to an ideal situation without sequencing error or bias. Because Cufflinks has a lot of parameters that are designed for real RNA-Seq experiments, we check that the conclusions drawn in section 3.1 are not too much influenced by this situation.

For that purpose we trained the methods on a first RNA-Seq data set before to run it on an independent test set with optimized parameters. In practice the training sets correspond to 1 million single-end or paired-end 150bp long reads simulated with the RNASeqReadSimulator software from 589 UCSC human transcripts on chromosome 18. The test sets are the ones described in section 3.1. Parameters of the simulator are unchanged between training and test data simulations.

We picked 10 parameters for Cufflinks denoted as *advanced assembly options* and tried 7 values for each of them (while other parameters fixed to their default values) equally distributed in log-space from default value divided by five to default value times five. This procedure is similar to the one explained in Behr *et al.* (2013). For FlipFlop we only optimized 2 parameters for the single-end experiments and 3 parameters for the paired-end ones. We kept for testing the parameters that optimized the F-score.

Figure S5 shows the best F-score obtained on the training sets for each parameter. Figure S6 shows precision and recall on the test sets when using either the parameter default values or the F-score optimized values. Cufflinks and FlipFlop performances are very similar when considering the F-score optimized case. However FlipFlop still shows quite better performances for 300bp long reads, suggesting that FlipFlop would be more appropriate for a real RNA-Seq experiment with such long reads, where it would not be possible to extensively tune parameters.

6 REALISTIC SIMULATIONS

We also performed more realistic simulations than the ones presented in section 3.1 of the main paper using the Flux-Simulator (Griebel *et al.*, 2012), which is a software designed to mimic a real RNA-Seq experiment workflow (fragmentation, reverse transcription, PCR amplification, filtering and sequencing). We generated 2 million 150bp long single-end reads from the 4140 UCSC human transcripts of multi-exons genes of chromosome 1. Note that we gave here to Cufflinks the fragment length mean and standard deviation, while FlipFlop does not need that information for single-end experiments. Moreover we performed two kinds of simulations, with or without GC bias during the PCR amplification step.

Precision and recall for Cufflinks and FlipFlop for the two experiments are shown in Figure S7. For both methods the inclusion of a GC bias affects the performance, but proportionally less for FlipFlop than for Cufflinks. Results with default parameters are shown in red, and for this particular set of experiments FlipFlop clearly outperforms Cufflinks both in precision and recall.

We also show FlipFlop's results when applying a GC correction during the isoform recovery process. It simply corresponds to multiplying each Poisson parameter of each bin by the GC content

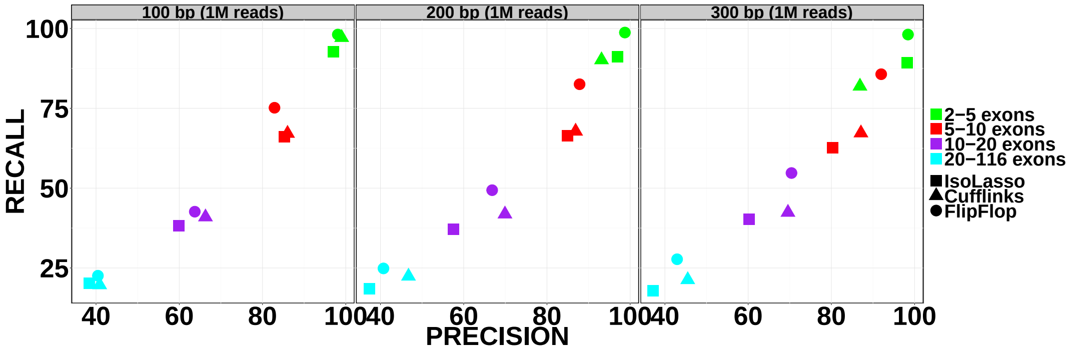

(a) Single-end reads with different lengths (100, 200, 300bp) and 1 million reads by exon level

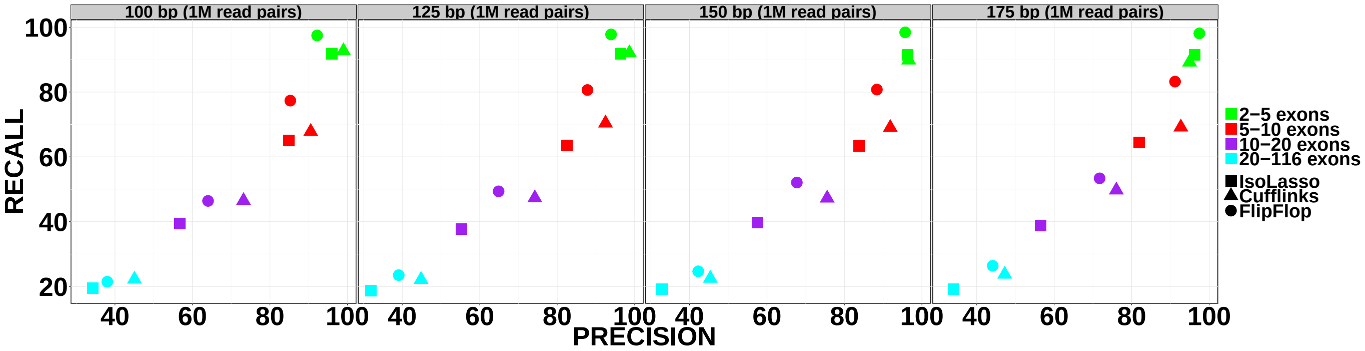

(b) Paired-end-end reads with different lengths (100, 125, 150, 175bp), 400bp mean fragment length and 1 million read pairs by exon level

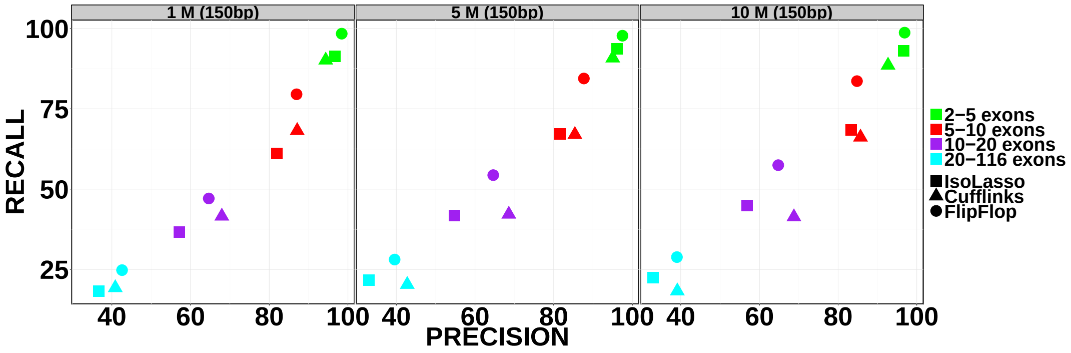

(c) Single-end reads with a fixed 150bp length and an increasing amount of material (1, 5, 10 million)

Fig. S3. Precision and recall on simulated reads from UCSC annotated human transcripts with an exon stratification.

of the bin. Using this correction slightly increases the accuracy of FlipFlop.

Finally we add FlipFlop's precision-recall curves, obtained when varying the BIC constant used for model selection (see section 2.6 of the main paper for more details about the model selection strategy). Surprisingly these curves have a bell shape: the recall increases first when the BIC constant decreases (light blue to dark blue colors) before to fall down for very small BIC constants. Using a small BIC constant corresponds to using a small regularization parameter λ in equation (2), and finally selecting a complex model with many isoforms. If the model is allowed to be very complex, several small isoforms are preferred to fewer long ones, and it might happen than some correct long isoforms are discarded from the solution. One way to deal with that problem in future work would be to penalize short isoforms by giving appropriate costs on the edges of the splicing graph.

7 REAL RNA-SEQ DATA

Section 3.2 of the main paper gives precision and recall on two human embryonic stem cell data sets. Figure S8 shows the running time of IsoLasso, Cufflinks and FlipFlop for these two experiments. Cufflinks and FlipFlop have similar running times on the paired-end experiment, while FlipFlop is a little bit faster on the single-end one and IsoLasso is much faster in both cases. While Figure 5 of the main paper shows precision and recall for different FPKM levels, Figure S9 consider all abundances and shows FlipFlop's

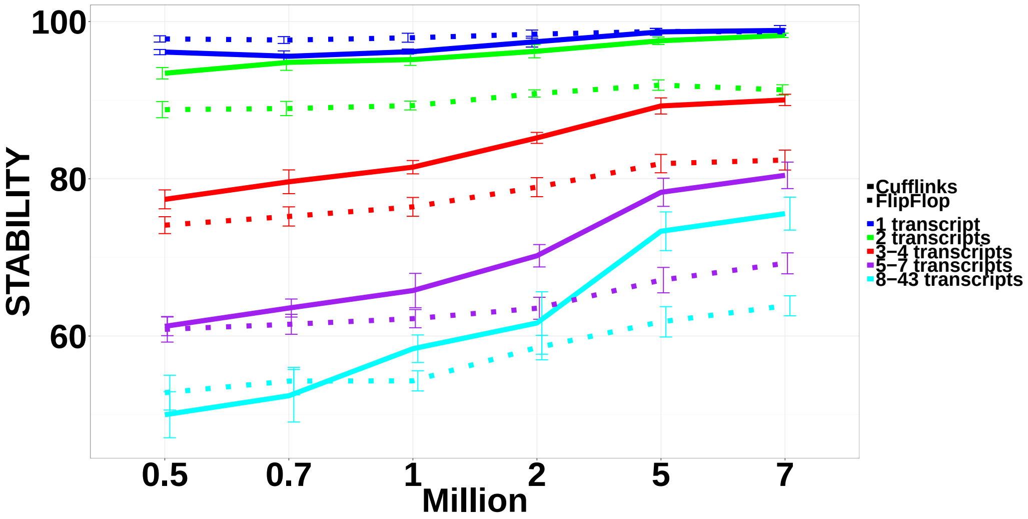

Fig. S4. Stability versus million of simulated 150bp long single-end reads from UCSC annotated human transcripts. Cufflinks corresponds to the solid line and FlipFlop to the dotted line.

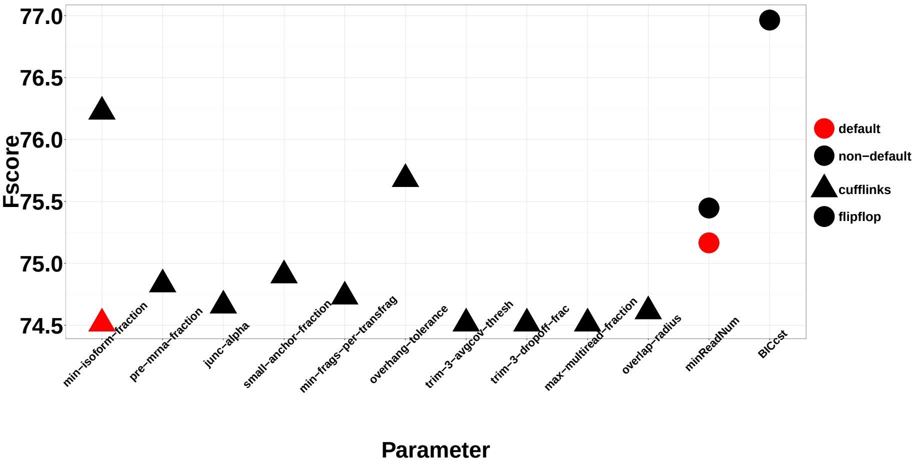

(a) Best F-score on the single-end reads training set

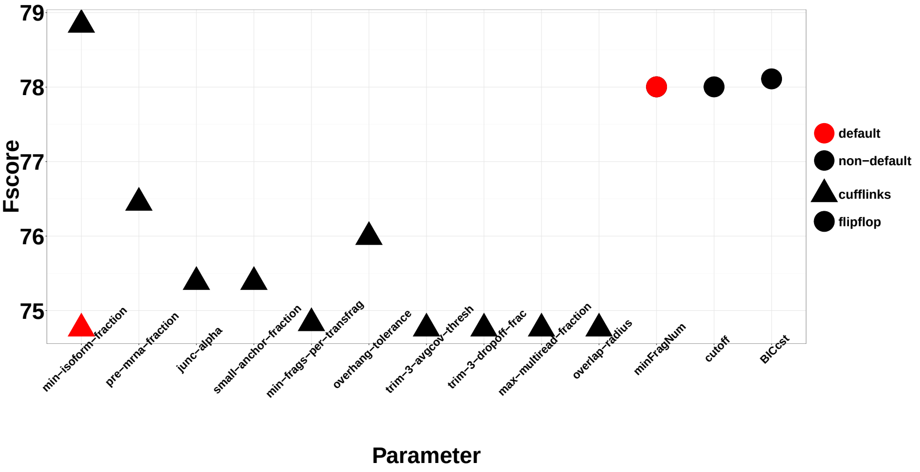

(b) Best F-score on the paired-end reads trainings set

Fig. S5. Best F-score obtained when varying parameters on the simulated training sets. F-score obtained with default parameters are in red.

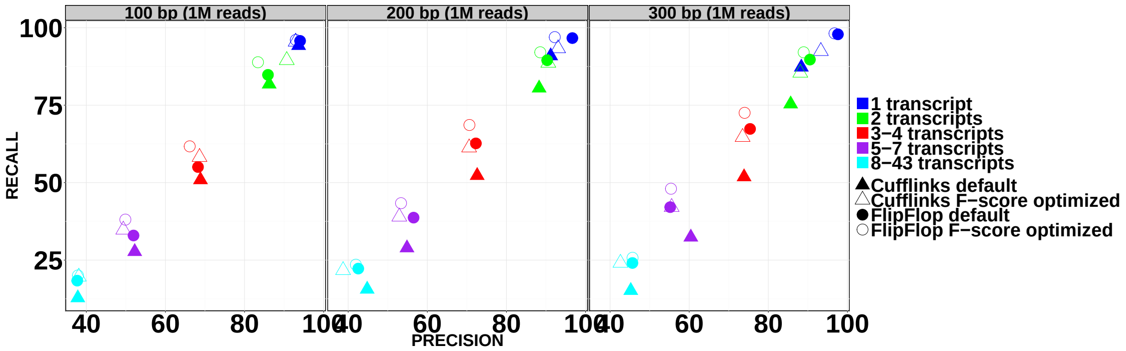

(a) Single-end reads with different lengths (100, 200, 300bp) and 1 million reads by transcript level

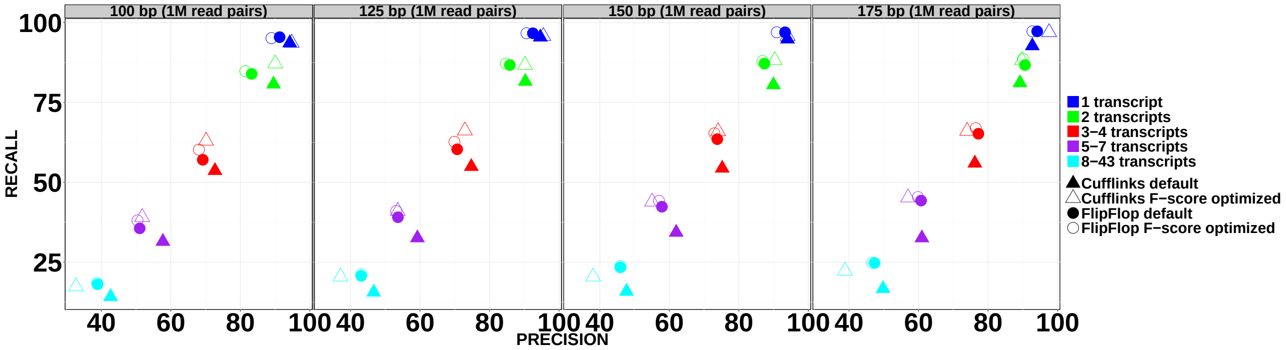

(b) Paired-end-end reads with different lengths (100, 125, 150, 175bp), 400bp mean fragment length and 1 million read pairs by transcript level

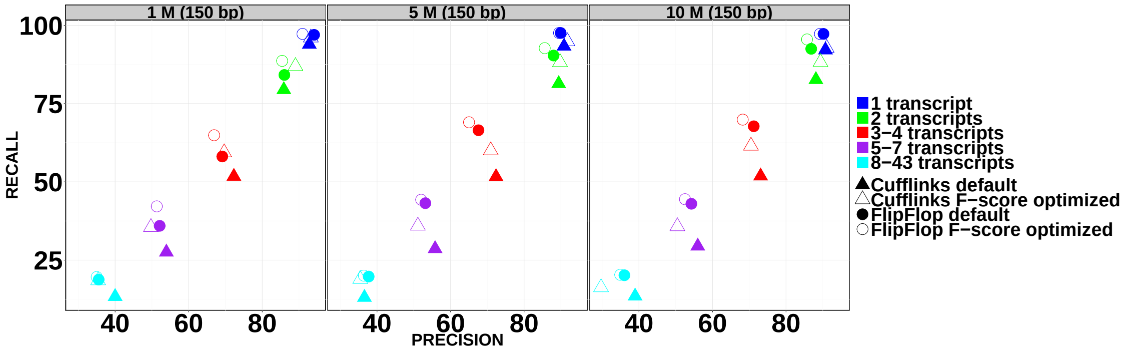

(c) Single-end reads with a fixed 150bp length and an increasing amount of material (1, 5, 10 million)

Fig. S6. Precision and recall on test sets for Cufflinks and FlipFlop with default or F-score optimized parameter values.

precision-recall curves obtained when varying the model selection rule. When using default parameters, IsoLasso and FlipFlop have similar precisions while FlipFlop has a 4% and 6% better recall in respectively the paired and single-end experiments. FlipFlop has a 9% higher precision than Cufflinks in both experiments while Cufflinks has a 5% and 2% better recall. In both cases, IsoLasso point is under the precision-recall curves, while Cufflinks point is above the curve on the paired-end case and on the curve on the single-end case. We also plan to try real RNA-Seq data with longer reads than 75bp.

REFERENCES

Behr, J. *et al.* (2013). Mitie: Simultaneous rna-seq based transcript identification and quantification in multiple samples. *Bioinformatics*, **29**, 2529–2538.

Griebel, T. *et al.* (2012). Modelling and simulating generic rna-seq experiments with the flux simulator. *Nucleic Acids Res*, **40**(20), 10073–10083.

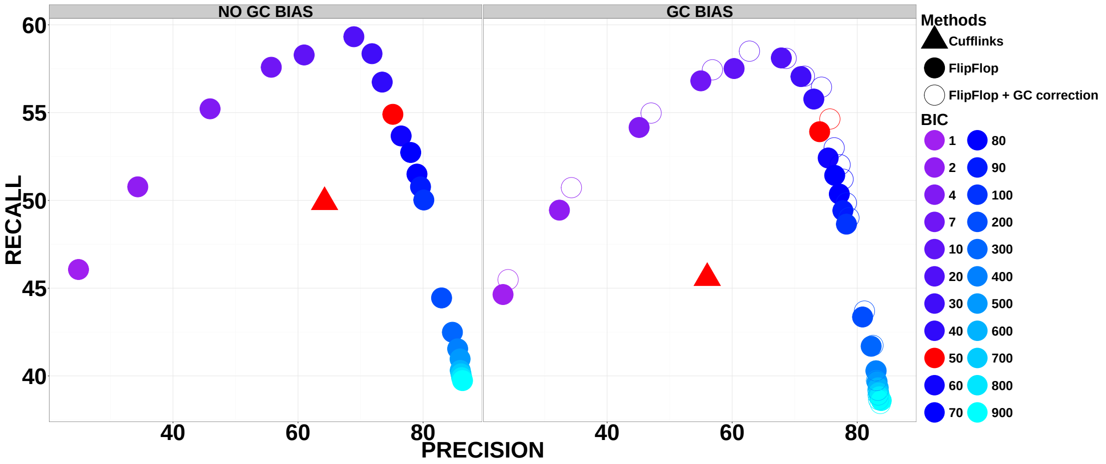

Fig. S7. Precision and recall on simulated reads with FluxSimulator from 4140 UCSC human transcripts. Results obtained with default parameters are in red.

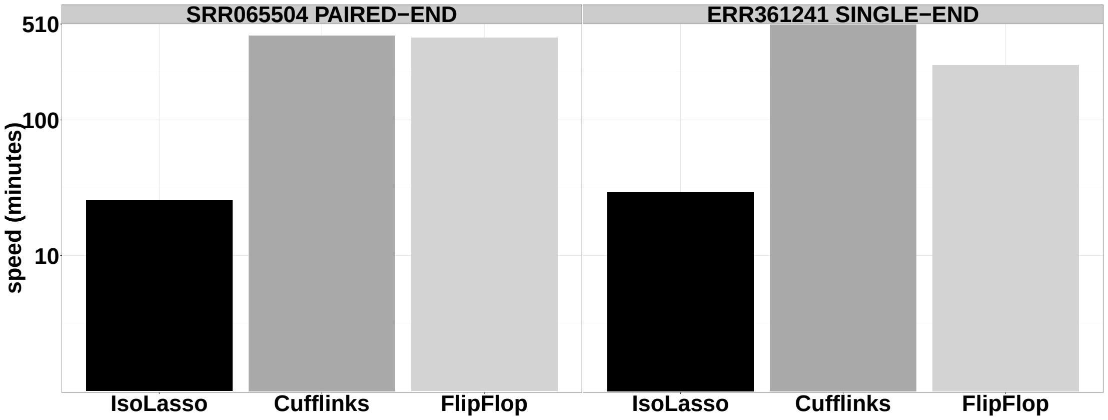

Fig. S8. Speed in minutes on a logarithmic scale of compared methods on human embryonic stem cells data.

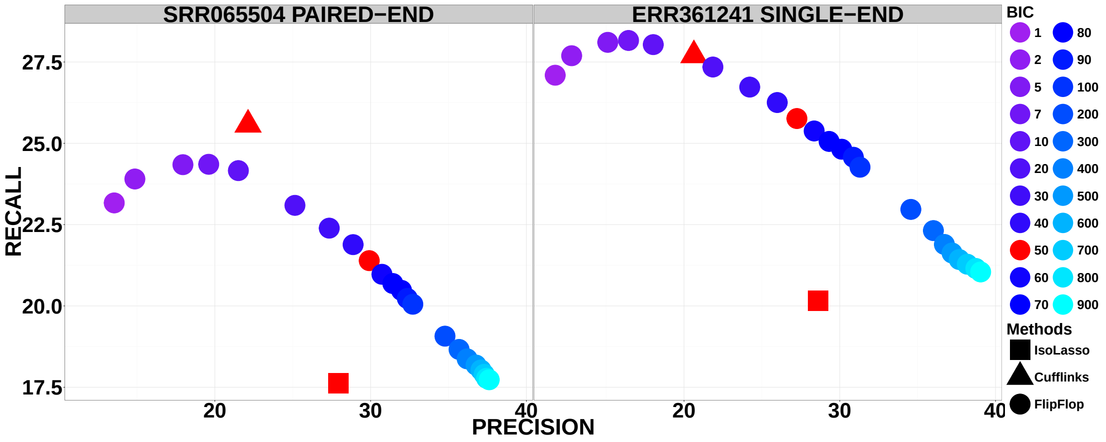

Fig. S9. Precision and recall on human embryonic stem cells. Results obtained with default parameters are in red.
